# RUNX1: A MicroRNA Hub in Normal and Malignant Hematopoiesis

**DOI:** 10.3390/ijms14011566

**Published:** 2013-01-14

**Authors:** Stefano Rossetti, Nicoletta Sacchi

**Affiliations:** Department of Cancer Genetics, Roswell Park Cancer Institute, Buffalo, NY 14263, USA; E-Mail: stefano.rossetti@roswellpark.org

**Keywords:** RUNX1 (AML1), microRNAs (miRNAs), hematopoiesis, leukemia, transcription factor-microRNA circuits, gene regulatory networks

## Abstract

Hematopoietic development is orchestrated by gene regulatory networks that progressively induce lineage-specific transcriptional programs. To guarantee the appropriate level of complexity, flexibility, and robustness, these networks rely on transcriptional and post-transcriptional circuits involving both transcription factors (TFs) and microRNAs (miRNAs). The focus of this review is on RUNX1 (AML1), a master hematopoietic transcription factor which is at the center of miRNA circuits necessary for both embryonic and post-natal hematopoiesis. Interference with components of these circuits can perturb RUNX1-controlled coding and non-coding transcriptional programs in leukemia.

## 1. Introduction

During embryonic and post-natal hematopoiesis, a limited pool of hematopoietic stem cells (HSCs) gives rise to all blood cell types through hierarchical specification of different hematopoietic lineages. Development, self-renewal, lineage commitment, and maturation of HSCs and multipotent progenitor cells are orchestrated by soluble growth factors and signals from the microenvironment, as well as cell-autonomous changes in gene expression. During hematopoietic differentiation, lineage-specific genes are progressively induced, while alternative-lineage genes are progressively silenced through heritable chromatin changes. The regulation of these processes relies on a complex interplay between hematopoietic transcription factor networks and post-transcriptional regulators, in particular microRNAs (miRNAs). In this review, we specifically focus on RUNX1, a master hematopoietic transcription factor at the center of a miRNA network relevant for both normal and malignant hematopoiesis.

## 2. Transcription Factor-MicroRNA Networks in Hematopoiesis

Hematopoiesis starts during embryonic development and continues throughout life to guarantee proper generation and constant availability of blood cells (for general reviews see [1–3]). During embryonic development, hematopoiesis occurs in two waves. In the first wave, hematopoiesis (called primitive) takes place in the yolk sack and results in the transient production of primitive hematopoietic cells, mainly erythrocytes, necessary to support initial embryonic growth [1]. These primitive cells are morphologically different from, and do not give rise to, adult hematopoietic cells. In the second wave, hematopoiesis (called definitive) occurs at multiple sites, and eventually gives rise to the adult hematopoietic system. During definitive hematopoiesis, a pool of hematopoietic stem cells (HSCs) is produced in both extra embryonic and embryonic regions derived from the mesoderm, such as the placenta and the aorta-gonad mesonephros (AGM) region [2]. In these regions, mesoderm-derived endothelial/blood precursors differentiate into a “hemogenic endothelium”, which undergoes an epithelial-hematopoietic transition whereby endothelial-like cells become non-adherent and acquire HSC features [2,4]. These HSCs later colonize the fetal liver and, ultimately, the bone marrow, where they sustain hematopoiesis throughout life. HSCs are capable of both self-renewal and generation of multipotent progenitor cells, which progressively become committed and produce distinct hematopoietic lineages [1,2].

The different hematopoietic cell types are characterized by very specific gene expression profiles that reflect the progressive restriction of their differentiation potential [5]. Several transcription factors (TF) play a key role in determining the correct temporal activation or repression of specific hematopoietic transcriptional programs during both HSC establishment/maintenance and lineage-specific differentiation and function (for general reviews see [1,5,6]). For instance, the ETS transcription factor FLI1 is indispensable for the formation of the epithelial/blood precursors [7], SCL/TAL1 is required for their differentiation into the hemogenic endothelium [8,9], and RUNX1 is necessary for the formation of HSCs from the hemogenic endothelium [10–12]. Differentiation into more committed progenitors and mature blood cells involves also other TFs, including NFI-A, PU.1 and CEBP family members, whose balance is critical to determine myeloid and lymphoid cell fates [13–21]. Interestingly, HSCs express not only TFs strictly required for their homeostasis and self-renewal, but also many others that characterize the development of multiple lineages [5]. Thus, during lineage commitment it is not only necessary to activate the gene networks that identify a specific lineage, but also to repress the programs associated with alternate lineages [5]. It is becoming apparent that this level of complexity cannot be determined by single TFs, but instead relies on positive and negative regulatory interactions within TF networks [22,23] as well as TF-miRNAs circuits [24–26].

The role of miRNAs in hematopoiesis is well established, and has been recently and amply reviewed [25–30]. One of the most remarkable properties of miRNAs is their ability to affect entire cellular processes by controlling multiple targets of the same pathway [31]. In this respect, very small changes in miRNA expression are expected to have a cumulative effect and result in much greater biological outcomes. The reach of miRNA action is further extended by the interplay between miRNA-controlled networks and TF networks. Not only TFs and miRNAs frequently share the same targets, but some miRNAs often target TFs that modulate their own transcription. This interplay results in transcriptional/post-transcriptional feedback and feed-forward loops that both expand and fine-tune the action of single TFs/miRNAs [31]. TF-miRNA circuits not only are predicted by bioinformatics’ analyses [32,33], but are also supported by growing experimental evidence, particularly from hematopoietic models (reviewed by [25,34]). For instance, monocytic and granulocytic differentiation involves at least three such circuits [25]. In one circuit, CEBPA (CCAAT/enhancer-binding protein alpha) is induced by granulocyte colony stimulating factor during granulopoiesis and is part of a feed-forward loop whereby CEBPA positively modulates miR-223 and NFIA transcription, while miR-223 represses NFIA expression [35]. Repression of NFIA on one hand prevents the initiation of erythropoietic transcriptional programs, and on the other hand drives granulopoiesis [20,21]. In a second circuit, PU.1 induces both miR-424 and NFI-A, while miR-424 represses NFIA expression [36]. Both NFI-A downregulation by RNAi and miR-424 overexpression have been shown to promote monocytic differentiation [36]. In addition, as we will examine more in detail later in this review, monocytic differentiation involves a third circuit linking RUNX1 and the miR-17-92 cluster [37].

Establishing transcriptional and post-transcriptional feedback circuits gives cells, at the same time, sufficient flexibility to instruct specific differentiation programs, and the necessary robustness to maintain these programs throughout cell life [23]. It is not surprising that interference with any of the components of these complex circuits often leads to hematopoietic malignancies [38].

## 3. RUNX1 in Hematopoiesis and Leukemia

### 3.1. RUNX1: A Master Regulator of Hematopoiesis

RUNX1 (also known as AML1 and CBF alpha) plays an essential role both in the generation of definitive HSCs during embryonic development and in the maintenance of lineage differentiation during adult hematopoiesis (for general reviews see [39–42]). RUNX1 was originally identified in acute myeloid leukemia (AML) patients with t(8;21), where the fusion with the ETO gene impairs its function [43–46]. During mouse development, Runx1 is expressed in specific subsets of endothelial cells in all embryonic and extraembryonic hematopoietic sites, even before the emergence of definitive HSCs, thus suggesting a critical role in the hemogenic endothelium [11,42]. Indeed, homozygous Runx1 knock out in mice is embryonic lethal due to extensive hemorrhages (derived from primitive erythroblasts) and complete absence of definitive hematopoietic progenitors and HSCs in all hematopoietic sites [47,48]. Surprisingly, mice in which Runx1 was conditionally knocked out in endothelial cells, displayed the same hematopoietic deficiencies observed in non-conditional Runx1 knockout mice, while mice with Runx1 conditional knock out in hematopoietic-committed cells still displayed definitive hematopoiesis [10]. This finding shows that Runx1 plays an essential role in the formation of HSCs from the hemogenic endothelium, but its expression is no longer necessary for embryonic hematopoiesis once HSCs have been established. Nevertheless, Runx1 widespread expression in most adult hematopoietic lineages and HSCs suggests a role also in post-natal hematopoiesis [49,50]. According to a few studies, conditional Runx1 knockout in adult HSCs results in expansion of the Lin-Sca-c-Kit+ population (putative HSCs), but the same effect was not observed in other studies [10,51–54]. A more consistent phenotype associated with conditional Runx1 knockout in adult HSCs is the expansion of myeloid progenitors, which may be due to a partial block of myeloid differentiation [52,53]. Runx1 deficient adult mice also showed impaired lymphoid and megakaryocytic differentiation, a reduced number of lymphoid progenitors, and thrombocytopenia [52,53]. Overall, Runx1 seems to play a critical role as a differentiation inducer: first it is necessary for “differentiation” of endothelial cells into HSCs, then it reduces HSCs self-renewal and promotes differentiation of the myeloid, lymphoid and megakaryocytic lineages [39].

RUNX1 function in hematopoiesis is determined by cell context-specific interactions with DNA, other TFs, and co-factors. The characterizing domain of RUNX1 is the N-terminal Runt homology domain (RHD), a conserved DNA binding domain necessary both for the recognition of the DNA consensus sequences 5′-PuACCPuCA-3′ [55], and for binding to the co-factor CBF beta (CBFB) [56,57]. CBFB does not interact with DNA directly, but its binding strongly enhances RUNX1 DNA-binding affinity and is necessary for RUNX1 function [56–58]. The heterodimer formed by RUNX1 (also called CBF alpha) and CBFB is often referred to as a single functional unit, known as Core Binding Factor (CBF). Cbfb knock out in mice almost completely phenocopies Runx1 knock out, resulting in ablation of definitive hematopoiesis and embryonic lethality [58,59]. The C-terminus of RUNX1 contains regulatory regions that mediate the interaction with either transcriptional activators or transcriptional co-repressors [60–62]. By recruiting chromatin modifying enzymes such as the histone acetylases P300, CBP and MOZ [60,63], histone deacetylases (via the co-repressors Sin3A and Groucho/TLE) [64–66], or the Polycomb Repressive complex PRC1 [67], RUNX1 can locally modify the chromatin of specific target genes and either facilitate or impede their transcription. The occurrence of activation or repression is apparently determined by the cell context, the promoter context, the local interaction with other TFs and, possibly also by RUNX1 post-translational modifications [39,40,68,69]. During HSC formation, RUNX1 transcriptional activation seems to be the predominant function [42]. Indeed, a recent study shows that at the onset of definitive hematopoiesis, RUNX1 orchestrates global reorganization of lineage-specific TF assemblies with a concomitant increase in histone acetylation at specific regulatory elements [70].

A number of direct target genes have been described to be regulated by RUNX1, both in hematopoietic stem cells/precursors and more mature blood cells. For instance, RUNX1 directly binds and activates PU.1 and CEBPA, two transcription factors critical for determination of the myeloid and lymphoid lineage from hematopoietic precursors [71,72]. In more committed cells, RUNX1 directly modulates the expression of multiple lineage-specific genes, including myeloid-specific growth factor signaling genes such as IL-3, GM-CSF, M-CSF receptor (CSF1R), and genes relevant for myeloid function, such as MPO [73–76]. In addition, as we will discuss later in this review, RUNX1 regulates microRNAs involved in the development of different lineages.

### 3.2. RUNX1 Perturbations in Leukemia

RUNX1 and CBFB are frequent targets of chromosomal abnormalities in hematopoietic malignancies, particularly in leukemia (also referred to as CBF leukemia or CBFL). RUNX1 is one of the most common targets of chromosomal translocations in acute leukemia [40]. Over 50 chromosome translocations affecting the RUNX1 gene on chromosome 21 and over 20 different partner genes have been described [40,77,78]. One of the most recurrent translocations involving RUNX1 is the t(8;21), which is found in 30%–40% of acute myeloid leukemia (AML) FAB-M2 [40,77]. The t(8;21) juxtaposes part of the RUNX1 gene with part of the ETO (MTG8/RUNX1T1) gene on chromosome 8, and results in the production of the chimeric protein RUNX1-ETO (AML1-ETO/AML1-MTG8/RUNX1-RUNX1T1) [43–46]. This fusion protein retains the N-terminal, DNA-binding RHD domain of RUNX1, but loses the C-terminal regulatory regions of RUNX1, which are replaced by the *C*-terminal functional domains of the ETO protein. ETO belongs to the MTG family of transcriptional co-repressors and contains four conserved domains (NHR regions) that mediate homo/heterodimerization with other MTG proteins as well as interaction with histone deacetylases or other co-repressors [79]. Interestingly, another member of the MTG family, MTG16, is also found fused to RUNX1 in AML with t(16;21) translocation, and the resulting fusion protein RUNX1-MTG16 (AML1-MTG16) shares most of the molecular features of RUNX1-ETO [79,80].

Several studies indicate that RUNX1-MTG fusion proteins act in part as dominant negatives over wild type RUNX1, as they competitively bind to RUNX1 consensus sequences of the same target genes, but they also bring about the repressive activities of the MTG moiety [81–84]. Indeed, RUNX1-MTG proteins induce repressive chromatin changes at both DNA and histone levels in the regulatory regions of many myeloid genes typically activated by RUNX1, including CSF1R, p14ARF, and p21CIP1 [85–88]. However, it has been reported that RUNX1-ETO can also upregulate the expression of some RUNX1-targets [89,90] as well as bind preferentially regions with duplicated RUNX1 consensus sequences [91], suggesting that the effects of this fusion protein may involve a gain of function in addition to the loss of RUNX1 function. Mouse models indeed support this hypothesis. Early RUNX1-ETO knock-in mouse models were embryonic lethal and recapitulated many, but not all, of the phenotypes observed in Runx1 KO mice [92,93]. Subsequent transgenic models in which RUNX1-ETO was expressed only in adult bone marrow overcame embryonic lethality, and showed an increased number of granulocyte/macrophage progenitors [94]. This phenotype was similar to the one observed in Runx1 conditional knockout mice, but no lymphocytopenia or thrombocytopenia was observed [94]. Common to all RUNX1-ETO animal models developed so far is the inability of the fusion protein to induce overt leukemia [94–96], unless additional mutations are present [97]. In line with these findings is the observation that up to 50% of pediatric patients with t(8;21) display the translocation already at birth, but overt leukemia occurs only years later [98]. Apparently, t(8;21) cells remain in a “pre-leukemic” state until additional hits trigger leukemic growth.

Similarly to RUNX1, CBFB is also a target of chromosome rearrangements in leukemia. Approximately 3%–10% of AML cases are characterized by inv(16) [38], which leads to the fusion of the CBFB gene with MYH11 (coding for the smooth muscle heavy chain) and the consequent production of the chimeric protein CBFB-MYH11 [99,100]. Like AML1-ETO, although through a different mechanism, CBFB-MYH11 acts as a dominant negative of the wild type RUNX1/CBFB complex and leads to deregulation of RUNX1-target genes [101]. Non-conditional transgenic expression of CBFB-MYH11 in mice results in embryonic lethality with a phenotype similar to Runx1/Cbfb knockout mice [102], while conditional expression in the bone marrow results in an increase of the HSC population and abnormal myeloid progenitors [103]. Differently from AML1-ETO, expression of CBFB-MYH11 seems sufficient to generate AML [103].

A significant number of AML cases without karyotypic abnormalities involving RUNX1 or CBFB can still have an impaired CBF function, due to other factors such as point mutations, deletions, or simply transcriptional or post-transcriptional downregulation. The identification of inherited mono-allelic RUNX1 intragenic deletions in familial platelet disorder (FPD), which is associated with a higher risk to develop AML, points to RUNX1 haploinsufficiency, and not only RUNX1 translocations, as a leukemia pre-disposing factor [104]. More recent large-scale sequencing studies have uncovered the occurrence of RUNX1 mutations in a significant percentage of AML and myelodysplastic syndrome (MDS) [105–107]. The most frequent mutations occur in the RUNX1 RHD domain and result in loss of the protein function [108]. It is interesting to note that different mutations can result in different biological effects, which sometimes show gain of function relatively to the simple loss of RUNX1 [39,109]. Although RUNX1 mutations do not seem to be sufficient to trigger leukemia [39], many of them are associated with poor outcome [110]. It has been estimated that the combination of RUNX1 mutations and chromosome rearrangements affecting RUNX1/CBFB may account for approximately 28% of all adult AML cases [108]. The dramatic biological consequences of RUNX1 hypomorphic mutations or monoallelic loss [111] indicate that RUNX1 dosage is indeed critical for normal hematopoiesis.

## 4. RUNX1: A MicroRNA Hub in Normal and Malignant Hematopoiesis

### 4.1. MicroRNAs Targeting RUNX1

**Figure 1:**
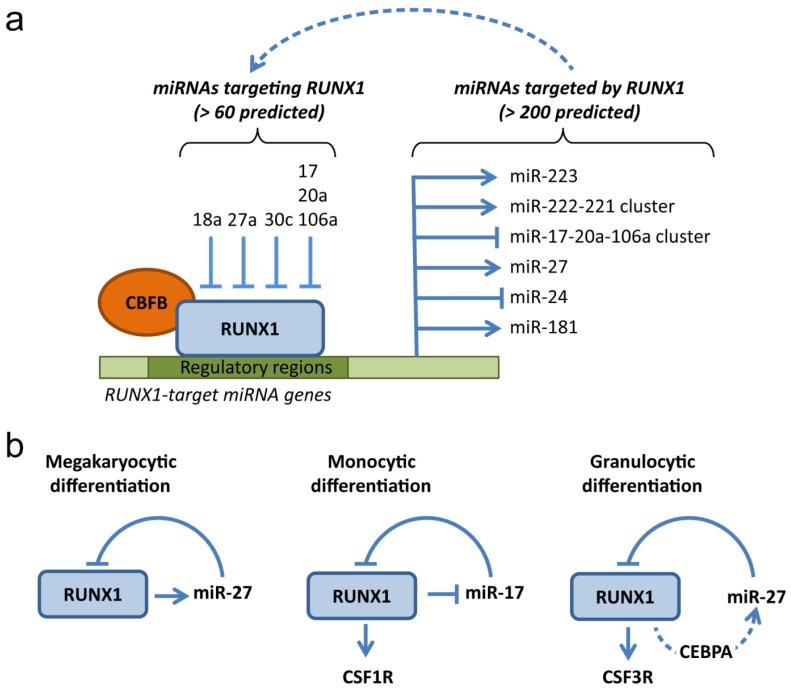
(**a**) RUNX1 is a hub of miRNAs targeting RUNX1 and miRNAs targeted by RUNX1. A number of miRNAs (>60 predicted by Targetscan, of which shown are only the ones experimentally validated) can inhibit RUNX1 protein expression by targeting the 3′UTR of RUNX1 mRNA. RUNX1 is predicted to target more than 200 miRNAs (shown are only the ones experimentally validated) and either repress or activate their transcription. Some miRNAs, such as miR-17 and miR-27, in turn can target RUNX1 in a feedback loop (dotted arrow). (**b**) Examples of possible RUNX1-miRNA feedback loops involved in megakaryocytic, monocytic, and granulocytic differentiation. In the latter case, RUNX1 may control miR-27 transcription indirectly, via CCAAT/enhancer-binding protein alpha (CEBPA) (dotted arrow).

RUNX1 is part of TF-miRNA circuits that guarantee a robust transcriptional and post-transcriptional control of its expression during hematopoiesis. RUNX1 expression is not only modulated by key hematopoietic TFs, such as GATA2, ETS factors, SCL [112] and by RUNX1 itself [113], but also by an expanding number of miRNAs. Basic in silico analyses with bioinformatics tools (e.g., TargetScan) predict over 60 conserved miRNAs targeting the longest RUNX1 3′UTR, many of which have been validated [37,114,115] (Figure 1). In particular, the miR-17-92 cluster and miR-27 seem to play an important role in regulating RUNX1 protein dosage and, consequently, RUNX1 function in hematopoietic differentiation.

The miR-17-92 cluster is transcribed into six distinct miRNAs, which include miR-17 and miR-20a [116]. MiR17-92 promotes cell proliferation and survival by targeting key tumor suppressors, such as p21CIP21 and PTEN [117,118], and is one of the first miRNAs with a validated oncogenic activity [119]. Indeed, miR-17 is frequently overexpressed in cancer, including lymphoma and leukemia [119–123]. Fontana *et al.* have shown that miR-17, miR-20a, and the highly homologous miR-106a, are expressed at high levels in early myeloid progenitors, but are downregulated during monocytic differentiation of human CD34+ cells [37]. All three miRNAs target conserved sites in the RUNX1 3′UTR, and their downregulation results in increased RUNX1 levels. RUNX1 upregulation, in turn, leads to increased transcription of its direct target gene for the macrophage colony stimulating factor receptor, CSF1R, which promotes macrophage differentiation [37]. Remarkably, the miR-17 and the miR-106 clusters contain RUNX1 consensus sequences in their promoter regions, and their transcription can be directly repressed by RUNX1 through a mutual negative feedback loop. This reciprocal inhibitory mechanism facilitates the switch from an undifferentiated state, in which high miR-17-106 levels maintain low levels of RUNX1 and CSF1R, to a differentiated state, where miR-17-106 levels decrease to allow RUNX1-mediated CSF1R upregulation [37] (Figure 1).

MiR-27 was identified as a candidate RUNX1-targeting miRNA through miRNA prediction algorithms, and further validated experimentally by two independent groups [114,115]. Ben-Ami *et al.* showed that miR-27a binds the 3′UTR of RUNX1, attenuating its expression [114]. Since miR-27a is transcriptionally regulated by RUNX1 in a feedback loop, the authors postulate that the upregulation of RUNX1 in the early hematopoietic stages positively regulates miR-27a to attenuate RUNX1 level during megakaryopoiesis. Interestingly, miR-27a increases upon induction of megakaryocytic differentiation of K562 cells, while it decreases during erythroid differentiation, suggesting a role in the determination of the erythroid/megakaryocytic lineages from the common precursor [114] (Figure 1). In addition, Feng *et al.* reported that miR-27 also plays a role in granulocytic differentiation [115]. During CSF3 (granulocyte colony stimulating factor)-induced granulocytic differentiation of 32D.cl3 cells, miR-27 is upregulated concomitantly with RUNX1 downregulation. Gain- and loss-of-function experiments showed that indeed miR-27 directly controls RUNX1 levels and affects granulocyte differentiation [115]. RUNX1 acts as a repressor of the CSF3 receptor (CSF3R) [90], and its downregulation by miR-27 would promote granulocytic differentiation by preventing RUNX1-mediated CSF3R repression. In this cell model, RUNX1 may not affect miR-27 level directly, but through regulation of CEBPA, a RUNX1-target TF that induces miR-27 transcription (Figure 1).

MicroRNA-mediated RUNX1 control can be modulated at multiple levels. First, miRNA action can be influenced by alternative splicing of the RUNX1 3′UTR. The RUNX1 gene encodes at least three splice variants, characterized by 3′UTRs that differ both in sequence and size. Splice variant 1 and 2 (AML1c and AML1b, respectively) share the same 3′UTR (over 4000 bp) and encode the longest RUNX1 protein isoforms, with similar structure and function. Splice variant 3 (AML1a) contains a very short 3′UTR (less than 400 bp) with a different sequence from the 3′UTR of the longer isoforms. This variant encodes for the shortest RUNX1 protein isoform, which lacks most of the longer RUNX1 functional domains. Since the long and short RUNX1 isoforms seem to have antagonistic effects on myeloid differentiation and proliferation [124], miRNAs could produce diverse biological responses by differentially regulating the level of the various RUNX1 isoforms. For instance, while miR-27 can target the 3′UTR of both short and long isoforms, even if with different repressive strength, miR-17 can target only the 3′UTR of the longer RUNX1 isoforms [114,115]. In addition, miRNA can affect RUNX1 dosage indirectly, by targeting TFs controlling RUNX1 transcription. This seems to be the case of miR-27, which targets GATA2 through a feedback loop [112,114]. Moreover, since RUNX1 can modulate its own transcription [113], miRNA-mediated RUNX1 post-transcriptional regulation could directly impact RUNX1 transcriptional control.

### 4.2. MicroRNAs Targeted by RUNX1

As we previously mentioned, RUNX1 directly modulates the transcription of entire coding gene networks through the recruitment of chromatin modifying enzymes. It has become more and more apparent that RUNX1 can similarly control miRNA genes endowed with RUNX1-consensus sequences in their regulatory regions. In two recent studies, RUNX1 occupancy was analyzed by ChIP-seq in different hematopoietic cells contexts [125,126]. A more in depth analysis of these ChIP-seq data reveals that a remarkable number (over 200 when two data sets are combined) of miRNA genes is physically bound by RUNX1. These studies suggest that RUNX1 regulates not only coding-gene networks, but also miRNA networks. Indeed, several RUNX1-target miRNAs have been identified and validated. These include the above mentioned miR-17 and miR-27, which are part of RUNX1-miRNA regulatory loops, as well as other miRNAs involved in hematopoietic differentiation and proliferation (Figure 1).

The first RUNX1-target miRNA, miR-223, was identified due to its deregulation by the RUNX1-ETO fusion protein [127]. In myeloid precursors, RUNX1 occupies a RUNX1-binding site in the miR-223 promoter and keeps the chromatin in a transcriptionally active state; in t(8;21)-positive cells, RUNX1-ETO competes with wild type RUNX1 for the miR-223 RUNX1-binding site and induces repressive chromatin modifications that silence the gene [127]. MiR-223 targets a number of genes critical for granulocyte function and development. Mutant mice lacking miR-223 display an increased number of granulocyte precursors, a phenotype that can be traced to the upregulation of the miR-223-target Mef2c, a transcription factor that promotes myeloid progenitor proliferation [128]. In addition, miR-223 is part of at least two TF-miRNA circuits that play a key role in granolupoiesis: the miR-223-CEBPA-NFIA circuit that we have previously mentioned [35], and a feedback loop involving the transcription factor E2F1 [129]. E2F1 is a miR-223 target that inhibits granulopoiesis and induces myeloid cell cycle progression [129]. Induction of miR-223 results in downregulation of E2F1, thus leading to inhibition of cell cycle progression followed by myeloid differentiation. Since E2F1 represses miR-223 transcription, its downregulation concurs to increase miR-223 levels in a self-reinforcing loop [129].

We recently found that RUNX1 is also a direct transcriptional regulator of the miR-222-221 cluster. MiR-221 and miR-222 are highly homologous and target, among others, the tyrosine kinase receptor KIT [130]. Upon binding to its ligand, the stem cell factor (SCF), KIT activates downstream signaling pathways involved in survival, proliferation, and differentiation [131]. KIT plays a key role in maintaining self-renewal of hematopoietic stem cells at all developmental stages [132]. During erythropoiesis, miR-222-221 expression declines, and leads to increased KIT protein levels and expansion of early erythropoietic cells [130]. Remarkably, KIT is often overexpressed in CBF leukemia concomitantly with miR-222-221 downregulation [133], suggesting that miR-222-221 may be under the transcriptional control of CBF (*i.e.*, RUNX1 and CBFB). Indeed RUNX1 binds to a conserved consensus sequence in the miR-222-221 promoter and induces its transcriptional activation [133]. Interestingly, KIT appears to be targeted also by miR-193 [134,135] and miR-494 [136], both of which contain RUNX1 consensus sequences in their promoter regions (unpublished observations).

RUNX1 targets also other miRNAs involved in hematopoietic proliferation and differentiation. For instance, RUNX1 donwregulates miR-181 [137], which seems to function as a molecular switch during hematopoietic lineage progression [138]. MiR-181 promotes megakaryocytic differentiation through repression of Lin28, and its inhibition has been shown to retard megakaryocytic differentiation in K562 cells [138]. Similarly, RUNX1 downregulates miR-24 [137], which plays a role in myeloid differentiation. MiR24 functions, at least in part, by downregulating MKP-7, a negative regulator of MAPK signaling. Consistently, miR-24 overexpression in myeloid progenitors results in a hyperproliferative phenotype and block of granulocytic differentiation [137]. MiR-24 is part of the miR-24-23-27 cluster, and RUNX1 directly interacts with consensus sequences in the promoter region of this cluster, repressing the transcription of the miR-24-23-27 pri-miRNA [137]. However, in another study, RUNX1 binding to consensus sequences in the miR-27 locus was instead associated with miR-27 upregulation [114]. Further studies are needed to clearly dissect how RUNX1 regulates pri-miRNA transcription as well as the level of the single miRNAs derived from this cluster.

Considering the number of miRNAs containing RUNX1-consensus sequences [125,126], it is likely that RUNX1 positively or negatively modulates the transcription of many other target miRNAs. Moreover, RUNX1-target transcription factors, such as CEBPA and PU.1, can also modulate the transcription of several other miRNAs relevant to hematopoiesis [139–142]. Thus, the range of RUNX1-mediated miRNA regulation could extend even further by encompassing not only direct miRNA targets, but also indirect miRNA targets via RUNX1-regulated TFs.

### 4.3. Deregulation of RUNX1-Related miRNAs in Leukemia

Both coding and miRNA genes transcriptionally controlled by RUNX1 are frequently deregulated in leukemia due to perturbation of RUNX1 function. As we briefly anticipated earlier in this review, the RUNX1-ETO fusion protein generated in t(8;21)-positive AML directly downregulates miR-223 through recruitment of histone deacetylases (HDACs) and DNA methyl transferases (DNMTs), which impose repressive chromatin changes in the miR-223 promoter [127]. MiR-223 downregulation is common in t(8;21) patients, and may directly contribute to leukemogenesis by preventing granulocyte differentiation and promoting myeloid progenitor proliferation [127,128]. Similarly, we found that RUNX1-MTG fusion proteins can directly repress miR-222-221 transcription through direct binding to conserved RUNX1 consensus sequences in the miR-222-221 promoter [133]. Consistently, this miRNA cluster is frequently downregulated in CBF leukemia patients, concomitantly with the upregulation of its target KIT [133]. Upregulation of KIT, in turn, confers to t(8;21) cells a proliferative advantage, thus facilitating the transition from a pre-leukemic state to overt leukemia. Through analogous mechanisms, CBF fusion proteins are expected to deregulate the expression of many other RUNX1-target miRNAs. For instance, close inspection of published miRNA profiling datasets shows that miR-181, which is downregulated by wild type RUNX1, is consistently upregulated in t(8;21) AML samples [120,137,143]. In addition, both miR-24 and miR-27 have been found upregulated in CBF leukemia samples [120,137], while miR-17 and miR-20a seem to be mostly downregulated [123,143]. Deregulation of RUNX1-target miRNAs, however, is often observed also in non-CBF leukemia samples, indicating that these miRNAs can be targeted by other TFs, and/or that factors other than cytogenetic abnormalities can affect RUNX1 function or expression.

**Figure 2:**
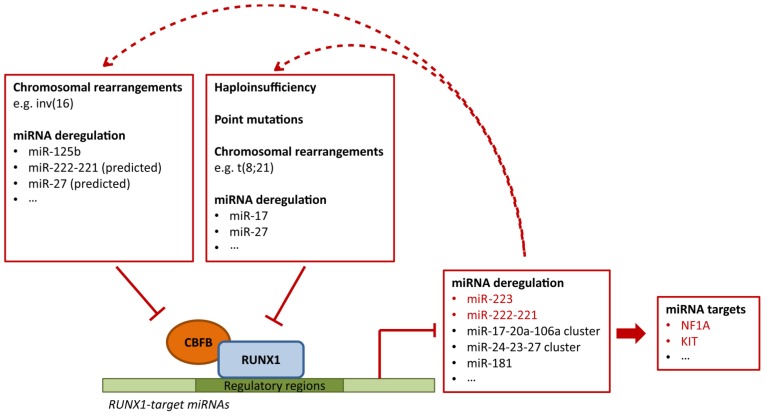
Several factors can potentially disrupt RUNX1-miRNA circuits, including deregulation of RUNX1-targeting miRNAs. RUNX1 function has been shown to be deregulated by altered RUNX1 dosage (haploinsufficiency), point mutations, or chromosomal rearrangements (e.g., t(8;21), producing RUNX1-ETO). In addition, RUNX1 expression could be affected by deregulation of miRNAs targeting RUNX1. The impairment of RUNX1 function would lead to deregulation of RUNX1-target genes, including miRNAs (in red are shown the RUNX1-target miRNAs known to be repressed by RUNX1-ETO, while in black are shown other established RUNX1-target miRNAs). Deregulation of miR-223 and miR-222-221 by RUNX1-ETO is known to affect their targets, NF1A and KIT. Since RUNX1 can target, and be targeted by, the same miRNAs, deregulation of wild type RUNX1 may be reinforced or weakened through a feedback loop (dotted arrow). Similarly, RUNX1 function could be affected by impairment of its heterodimeric partner CBFB, which could be subjected to miRNA-mediated deregulation in addition to known chromosomal rearrangements.

It is likely that other factors in addition to chromosome rearrangements can affect RUNX1-target miRNAs. In this respect, it is particularly intriguing that deregulation of miRNA *targeting* RUNX1 may result in deregulation of miRNA *targeted* by RUNX1. Since some of these miRNAs act in feedback loops, the deregulation of RUNX1 could either reinforce or counteract itself according to the nature of the loop (positive or negative feedback). For example, miR-17 upregulation, frequent in MLL-rearranged acute leukemia [123], would reduce RUNX1 levels and result in decreased RUNX1-mediated miR-17 transcriptional repression. This feedback loop would reinforce miR-17 upregulation and further impair RUNX1 expression. In contrast, overexpression of miR-27, by decreasing RUNX1, would lead to its own transcriptional downregulation. It is of note that miRNAs can affect RUNX1 function also by targeting CBFB. A notable example is miR-125b, which plays an important role in granulocyte differentiation and is often deregulated in hematopoietic malignancies ([144,145]). In addition, in silico analyses predict that many miRNAs (e.g., miR-27) can concomitantly target RUNX1 and CBFB, and that some RUNX1-target miRNAs (e.g., miR-222-221) could affect their own transcription by targeting CBFB (Figure 2).

MiRNA-based deregulation of RUNX1 expression could explain why non-CBF leukemias are often characterized by molecular defects, such as KIT upregulation, typically produced by CBF karyotypic abnormalities. However, decreasing RUNX1 expression may not necessarily recapitulate all the molecular and biological effects of CBF fusion proteins. Indeed, CBF fusion proteins not only act as RUNX1 dominant negatives, but display also gain of function properties, which could be due, for instance, to a different subcellular localization [137], or preferential binding to specific consensus sequences [91]. In addition, since miRNA action can differentially affect RUNX1 isoforms depending on their 3′UTR, miRNA deregulation could yield different biological effects according to the RUNX1 isoforms targeted. It is also interesting to note that the loss of the RUNX1 3′UTR in some RUNX1 fusion genes (e.g., RUNX1-ETO), and not in others (e.g., TEL-RUNX1), should differentially affect their post-transcriptional regulation by RUNX1-targeting miRNAs, and could contribute to define their oncogenic function.

## 5. Concluding Remarks

Gene regulatory networks (GRNs) have evolved in different organisms to guarantee the appropriate level of complexity, flexibility, and robustness necessary for development and cellular homeostasis [23]. GRNs typically have a modular and hierarchical structure, in which conserved smaller networks, or sub-circuits, with a given molecular/cellular function are combined to orchestrate cell fate [23]. Some of the best examples of these sub-circuits are represented by hematopoietic TF-miRNA circuits, such as the one centered on RUNX1. RUNX1 is part of circuits involving both RUNX1-targeting and RUNX1-targeted miRNAs. The involvement of RUNX1 in different stages of hematopoiesis suggests that RUNX1-based miRNA-TF circuits represent basic modules in developmental GRNs, and that these modules have the necessary versatility to be combined with other modules to direct the differentiation of distinct hematopoietic lineages. The centrality of RUNX1 circuits in hematopoiesis is further supported by the dramatic biological effects consequent to RUNX1 perturbation in hematopoietic malignancies (e.g., leukemia).

RUNX1-centered regulatory circuits may not be restricted to hematopoiesis. A growing number of studies report that RUNX1 is deregulated also in solid tumors, suggesting that RUNX1 may play a role in development/homeostasis of non-hematopoietic tissues [146–149]. In particular, RUNX1 seems to play an important role in breast acinar morphogenesis [150], and is frequently downregulated or mutated in breast cancer [149,151–154]. The role of RUNX1 in breast acinar development may be linked to estrogen receptor alpha (ERA) signaling. A recent genome-wide analysis has shown that prior to estradiol stimulation, RUNX1 is present at many ERA-binding sites to keep the chromatin in a permissive state for subsequent ERA recruitment [155]. Interestingly, miR-222-221 is activated by RUNX1 and repressed by ERA [133,156], suggesting an intertwined mechanism involving ERA, RUNX1, and specific miRNAs in breast cells.

In summary, RUNX1 seems to be a relevant hub of miRNA-regulated cell-specific circuits with diverse roles in multiple aspects of differentiation and development. Thus, deregulation of RUNX1-miRNA circuits is expected to be critical not only in the pathogenesis of leukemia, but also of other malignancies.
